# Analysis of individual and combined estrogenic effects of bisphenol, nonylphenol and diethylstilbestrol in immature rats with mathematical models

**DOI:** 10.1186/s12199-019-0789-5

**Published:** 2019-05-13

**Authors:** Weifeng Mao, Yan Song, Haixia Sui, Pei Cao, Zhaoping Liu

**Affiliations:** 1 National Institute for Nutrition and Health, Chinese Center for Disease Control and Prevention, No 27, Nanwei Road, Xicheng District, Beijing, 100050 China; 2 China National Center for Food Safety Risk Assessment, No 37, Building 2, Guangqu Road, Chaoyang District, Beijing, 100021 China

**Keywords:** Endocrine disrupting chemicals, Combined effects, Mixture toxicity, Factorial analysis, Concentration addition

## Abstract

**Background:**

Traditional toxicological studies focus on individual compounds. However, this single-compound approach neglects the fact that the mixture exposed to human may act additively or synergistically to induce greater toxicity than the single compounds exposure due to their similarities in the mode of action and targets. Mixture effects can occur even when all mixture components are present at levels that individually do not produce observable effects. So the individual chemical effect thresholds do not necessarily protect against combination effects, an understanding of the rules governing the interactive effects in mixtures is needed. The aim of the study was to test and analyze the individual and combined estrogenic effects of a mixture of three endocrine disrupting chemicals (EDCs), bisphenol A (BPA), nonylphenol (NP) and diethylstilbestrol (DES) in immature rats with mathematical models.

**Method:**

In the present study, the data of individual estrogenic effects of BPA, NP and DES were obtained in uterotrophic bioassay respectively, the reference points for BPA, NP and DES were derived from the dose-response ralationship by using the traditional no observed adverse effect (NOAEL) or lowest observed adverse effect level (LOAEL) methods, and the benchmark dose (BMD) method. Then LOAEL values and the benchmark dose lower confidence limit (BMDL_10_) of single EDCs as the dose design basis for the study of the combined action pattern. Mixed prediction models, the 3 × 2 factorial design model and the concentration addition (CA) model, were employed to analyze the combined estrogenic effect of the three EDCs.

**Results:**

From the dose-response relationship of estrogenic effects of BPA, NP and DES in the model of the prepuberty rats, the BMDL_10_(NOAEL) of the estrogenic effects of BPA, NP and DES were 90(120) mg/kg body weight, 6 mg/kg body weight and 0.10(0.25) μg/kg body weight, and the LOAEL of the the estrogenic effects of three EDCs were 240 mg/kg body weight, 15 mg/kg body weight and 0.50 μg/kg body weight, respectively. At BMDL_10_ doses based on the CA concept and the factorial analysis, the mode of combined effects of the three EDCs were dose addition. Mixtures in LOAEL doses, NP and DES combined effects on rat uterine/body weight ratio indicates antagonistic based on the CA concept but additive based on the factorial analysis. Combined effects of other mixtures are all additive by using the two models.

**Conclusion:**

Our results showed that CA model provide more accurate results than the factorial analysis, the mode of combined effects of the three EDCs were dose addition, except mixtures in LOAEL doses, NP and DES combined effects indicates antagonistic effects based on the CA model but additive based on the factorial analysis. In particular, BPA and NP produced combination effects that are larger than the effect of each mixture component applied separately at BMDL doses, which show that additivity is important in the assessment of chemicals with estrogenic effects. The use of BMDL as point of departure in risk assessment may lead to underestimation of risk, and a more balanced approach should be considered in risk assessment.

## Background

In the past few years, the need to evaluate the toxicity of endocrine disrupting chemicals (EDCs) mixtures has raised research concern [1, 2]. EDCs are a large group of chemicals (e.g., estrogenic, anti-androgenic or thyroid-disrupting agents) that are present as mixtures not only in water, soil, air and food, but also in humans. They can cause adverse health effects by interfering with the synthesis, secretion, transport, metabolism, binding action, or elimination of natural blood borne hormones in the body which are responsible for homeostasis, reproduction, and developmental process [2–4] . Traditional toxicological studies focus on individual compounds [5] In general, the exposure level of a single EDC is low and, so far, has not been shown to contribute to adverse human effects [6, 7]. However, this single-compound approach neglects the fact that the mixture of EDCs exposed to human may act additively or synergistically to induce greater toxicity than the single EDCs exposure due to their similarities in the mode of action and targets [8]. Mixture effects can occur even when all mixture components are present at levels that individually do not produce observable effects, and human disorders are more likely the result of chronic exposure to low amounts of mixtures of EDCs [9]. The traditional focus of risk assessment on single chemical is shifting toward considering combination effects of mixture chemicals [10], the individual chemical effect thresholds do not necessarily protect against combination effects [11], an understanding of the rules governing the interactive effects in mixtures is needed [12].

To address this need, we selected three well-known synthetic exogenous estrogen-like chemicals for study: bisphenol A (BPA), nonylphenol (NP), and diethylstilbestrol (DES). All three chemicals are a major concern for animal and human health due to their estrogenic effects and demonstrated human exposure [13–15]. Although several studies have described the potent estrogenic effects of BPA, NP, and DES in vitro and in vivo [6, 13, 14], the estrogenic effects of combined exposure to BAP, NP, and DES using in vivo models has not been adequately explored. Moreover, most EDC mixture studies used the direct effect addition method, which is problematic in that it simply added the effect of several individual chemicals to obtain a combined toxicity [16, 17]. The risk assessments of these EDCs by regulatory authorities have been based on the assumption that consumers are exposed to only one chemical at a time. Therefore, it is crucial to explore whether these xenoestrogens can act together to yield measurable responses when combined at concentrations that individually produce undetectable effects and whether such a combined intake of BAP, NP, and DES would lead to a possible higher risk of adverse health effects than the intake of one of these EDCs alone.

From the perspective of public health and chemical regulations, no observed adverse effect level (NOAEL), the benchmark dose (BMD) or the lowest observed adverse effect level (LOAEL) are used to define point of departure (POD) for dose-responses, which is used as the starting point of quantitative risk assessment. The POD is then combined with a safety factor to derive a health-based guidance value, e.g. acceptable daily intake (ADI) or tolerable daily intakes (TDI) [18]. When exposured to a large number of EDCs, the question is whether this claim is tenable for EDCs around their TDI. In this study, we presented the data of estrogenic properties of BPA, NP and DES in uterotrophic bioassy, respectively, and gained the benchmark dose lower confidence limit (BMDL_10_) and LOAEL values of single BPA, NP and DES by dose-response relationship of uterus/body weight ratio. Then we selected the BMDL_10_ and LOAEL level of each EDCs as the dose design basis for the study of the combined action pattern. And we will use the statistical analysis of factorial design method and dose addition model to examine whether the mode of combined effects of three EDCs is synergistic, antagonistic or additive, then provide scientific basis for further cumulative of risk assessment of these three EDCs.

## Methods

### Chemicals

BPA, NP and DES (≥99.0% purity) were purchased from Sigma Aldrich trading Co. LTD. (Shanghai, China). Corn germ oil was purchased from Fengyi trade Co. LTD. (Beijing, China). Radioactive kits of luteinizing hormone (LH), progesterone and estradiol (E2) were provided by the Institute of North Biological Engineering Research (Beijing, China). Soy free diet was provided by the Hua Fu Kang Biotechnology Co. LTD. (Beijing, China).

### Animals and exposure

Specific pathogen-free Sprague-Dawley (SD) rats (post-natal day 17d; body weight 28–33 g) were purchased from Hua Fu Kang Bioscience (Beijing, China). The animals were housed in cages under condition of controlled temperature (20–26 °C) and relative humidity (50%–65%), a 12 h light/12 h dark cycle and air change of 10 times/h. All animals were fed soy free diet (Hua Fu Kang Bioscience, Beijing, China) and provided with unlimited purified water throughout the study. All experiments are carried out in accordance with the Guide for the Care and Use of the Animals Management Rules of the Ministry Health of the People’s Republic of China (Documentation NO. 55, 2001, China). The study was approved by the Institutional Animal Care and Use Committee of China National Center for Food Safety Risk Assessment. During the experiment, all animals were treated humanely and maximum care was taken to minimize animal sufferings.

**Fig. 1:**
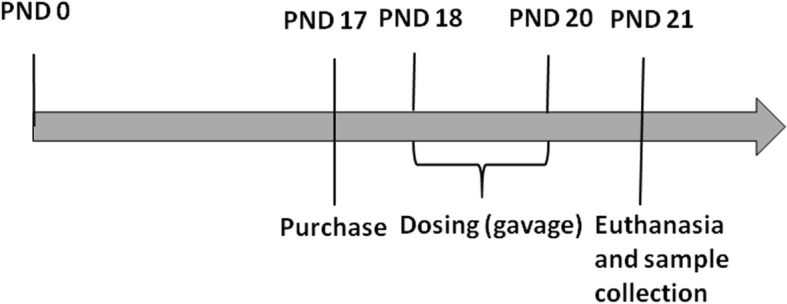
Schematic of the experimental design adopted for soes-response analyses of the individual chemicals and their mixtures. PND was the day of birth

This experiment was a uterotrophic bioassy in accordance with OECD Testing Guideline No. 440 [19](Fig. 1). Before conducting the mixture experiment, uterotrophic bioassays for each individual chemical were performed. 136 SD rats were randomly assigned into 17 groups (*n* = 8 per group) according to the body weight. The control groups consisted of untreated rats (blank) and solvent-treated rats (vehicle control). Five other groups received BPA at 15, 30, 60, 120, and 240 mg/kg body weight. Five other groups received NP at the same concentrations. The five other groups received DES at 0.25, 0.50, 1.0, 2.0 and 4.0 μg/kg body weight. All chemicals were dissolved in corn oil and administered orally via gavage once per day for 3 consecutive days. It aimed to find out the NOAEL, BMDL_10_ and LOAEL of estrogenic effects for individual chemical to determine the doses of mixture experiment.

For the mixture experiment, 160 SD rats were randomly assigned into 16 groups (*n* = 10 per group) according to the body weight. we selected BMDL_10_ and LOAEL of each chemical as the dose design basis for the study of the combined action pattern. All animals received EDCs exposure based on the dosimetry in Table 1 via gavage for 3 consecutive days.

**Table 1:** Dose combination of DES, BAP and NP

Group	*n*	Mixture Doses (mg/kg body weight)
blank control	10	water
vehicle control	10	Corn oil
BMDL_10_		
BPA	10	92
NP	10	6
DES	10	0.1
BPA + NP	10	92 + 6
BPA + DES	10	92 + 0.1
NP + DES	10	6 + 0.1
BPA + NP + DES	10	92 + 6 + 0.1
LOAEL		
BPA	10	240
NP	10	15
DES	10	0.5
BPA + NP	10	240+ 15
BPA + DES	10	240+ 0.5
NP + DES	10	15 + 0.5
BPA + NP + DES	10	240+ 15+ 0.5

*BPA* Bisphenol A, *NP* Nonylphenol, *DES* Diethylstilbestrol, *BMDL* Benchmark dose lower confidence limit, *LOAEL* Lowest observed adverse effect level

### Tissue weighing and fixing

The day after last gavage, all animals were fasted for 12 h and sacrificed under sodium pentobarbital anesthesia. Body weight and uterus wet weights were determined. The uterus of individual animals were preserved in 4% formaldehyde solution after weighing, and the uterus/body weight ratio was calculated according to uterus wet weight divided by body weight.

### Measurement of serum estrogen hormone levels

Blood was collected from ophthalmic vein in pro-coagulation tubes and centrifuged at 3000 rpm for 10 min to obtain blood serum and stored at − 80 °C until use. Serum luteinizing hormone (LH), progesterone and estradiol (E_2_) were determined by radioimmunoassay. All experiments were carried out according to the instructions of the manufacturer.

### Histopathology

At the same time, tissue recovered from the necropsy were fixed in 10% formalin, embedded in paraffin, sectioned, and stained with hematoxylin and eosin (HE) for histological examination using standard techniques. After hematoxylin & eosin staining, the slides were observed and photos were taken using optical microscope (Olympus X71,Japan). All the identity and analysis of the pathology slides were blind to the pathologist.

### Statistical analysis and BMD analysis

The “Statistical Product and Service Solutions (SPSS), version 19.0” (IBM Inc., Chicago, IL, USA) software was employed to compare the statistical differences between groups and conduct factorial analysis. BMDL were obtained based on the dose-response models from using “Benchmark Dose Software (BMDS), version 2.6” (Environmental Protection Agency, Washington D.C., USA).

### Factorial analysis

The mixture experiment was based on 2 × 2 × 2 factorial analysis of variance (ANOVA) [20]. The dose levels of each test substance were 0 mg/kg body weight, BMDL_10_ and LOAEL in trial test. The indexe in factorial design was the uterus/body weight ratio.

The original data were first analyzed for homogeneity of variance. If the data were heterogeneous, ANOVA could be conducted directly; otherwise logarithmic transformation was performed before ANOVA. As to main effects, a significant result (*P <* 0.05) indicated an effect of dose. As to combined effects, a significant result (*P <* 0.05) meaned the interaction effects exist among the test substances; otherwise, the combined effects would be additive.

If an interaction effect existed, corresponding skeleton map was used to decide if it’s synergistic effect or antagonistic effect. When antagonistic effects happened, the two lines in skeleton map would approach to each other, if synergistic effects existed, they would detach from each other.

### Mixture design

On the basis of the results for the individual compounds, a binary and a 3-compound mixture were made for hepatocyte bioassays. As the compounds were expected to act by similar mode of action, the mixture design was chosen on basis of the concept of CA. The model is defined for mixtures by equal to 1:

$$ {EC}_{x, mix}={\left(\sum \limits_{i=1}^n\frac{P_i}{EC_{xi}}\right)}^{-1} $$

where *EC*_*x*, *mix*_ is the total concentration of the mixture that causes x effect, *P*_*i*_ indicates the proportion of component i in the mixture, and *EC*_*xi*_ indicates the concentration of component i that would cause x effect [21–23].

Quantitative comparison of the observed toxicity and the models tested was conducted using a model deviation ratio (MDR). The ratio was calculated as the predicted effective concentration divided by the observed effective concentration. A MDR of 1 indicates that the mixture acts by additivity, a MDR > 1 indicates that the model underestimates the toxicity of the model, whereas a MDR < 1 indicates overestimation of toxicity by the model. However, mixtures with MDRs within a factor of 2 ((0.5 ≤ MDR ≤ 2)) are most likely to follow the concept of addition as this factor is within the expected interlaboratory/interexperiment deviation for most species [24]. So to evaluate the frequency of chemicals, chemical mixtures and species groups involved in synergistic (MDR > 2), additive (0.5 ≤ MDR ≤ 2) and antagonistic (MDR < 0.5) mixture experiments [25].

## Results

### Single chemical toxcity

#### Body weight and the uterus/body weight ratio

**Fig. 2:**
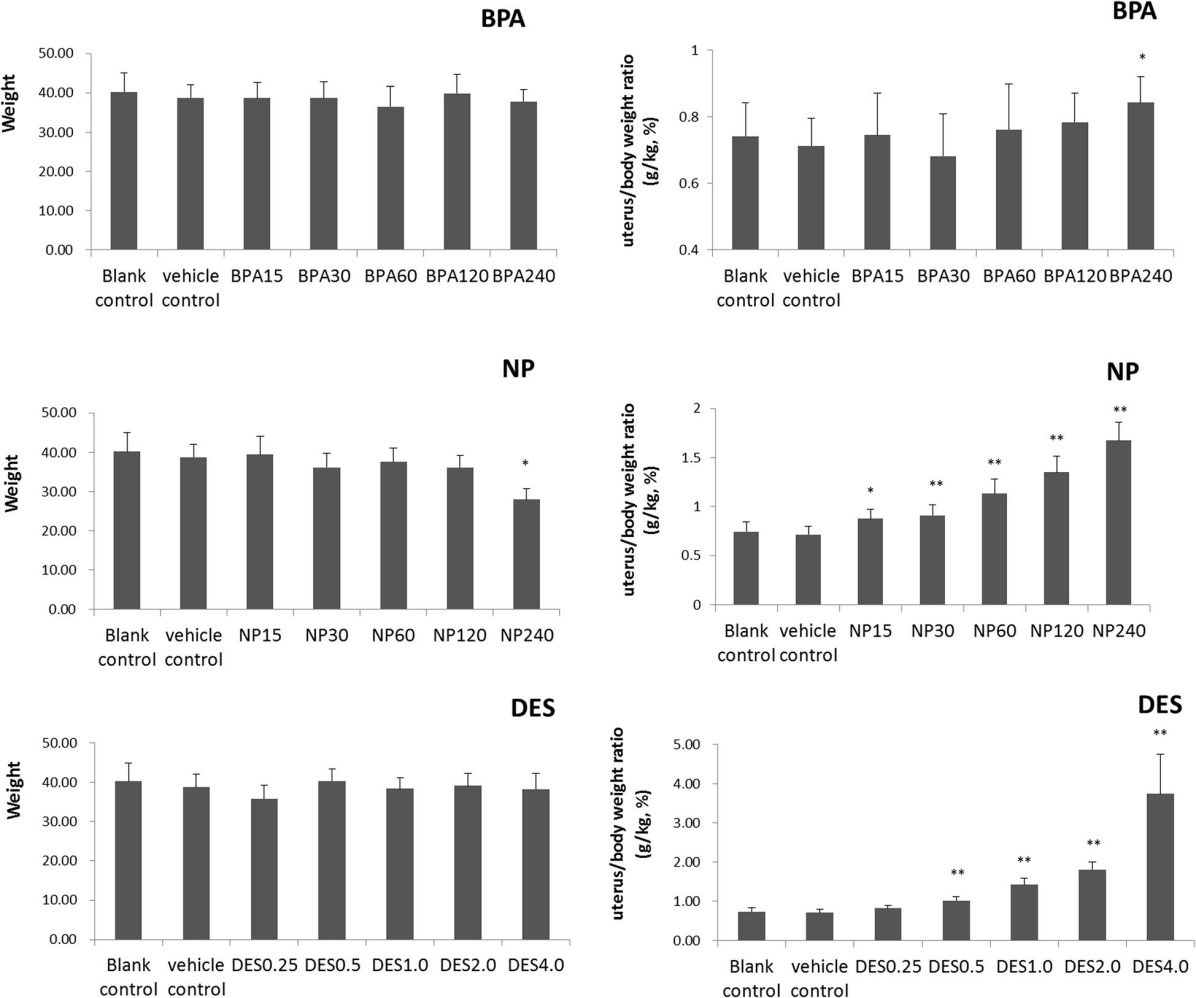
Weight and uterus/body weight ratio of rats receiving the different chemicals. *:*P* < 0.05; **:*P* < 0.01

According to body weight, no obvious changes were evident, except for the NP 240 mg/kg body weight group (Fig. 2). Compared to the vehicle group, the ratio of uterus/body weight in all NP groups, DES doses of 0.50, 1.00, 2.00, and 4.00 μg/kg body weight, and BPA dose of 240 mg/kg body weight were significantly increased (all *P* < 0.05). Thus, a significant dose-response relationship was evident for the three agents.

#### Serum LH, progesterone and E2 levels

**Fig. 3:**
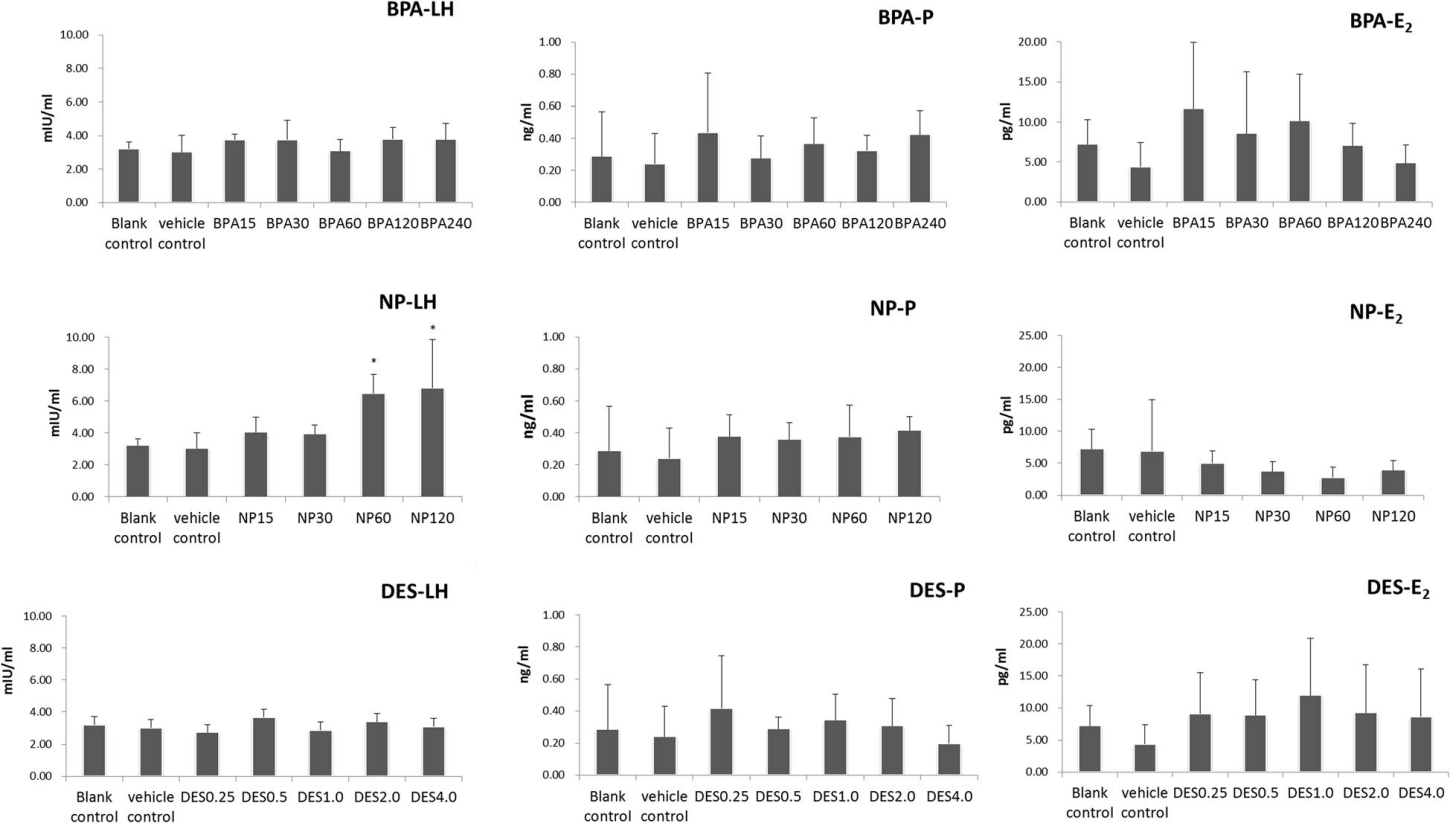
Effect of BPA, NP, and DES on LH, progesterone, and E2 in immature rats. *:*P* < 0.05

Figure 3 depicts the effects of BPA, NP, and DES on the serum concentration of LH, progesterone, and E2 in immature rats. Compared with the blank and solvent controls, LH was significantly increased in rats treated with NP at dosages of 120 and 240 mg/kg body weight (both *P* < 0.05). No obvious difference was apparent in the other groups. The concentrations of progesterone and E_2_ were not changed by any dosage of BPA, NP, and DES.

#### Pathological examinations

**Fig. 4:**
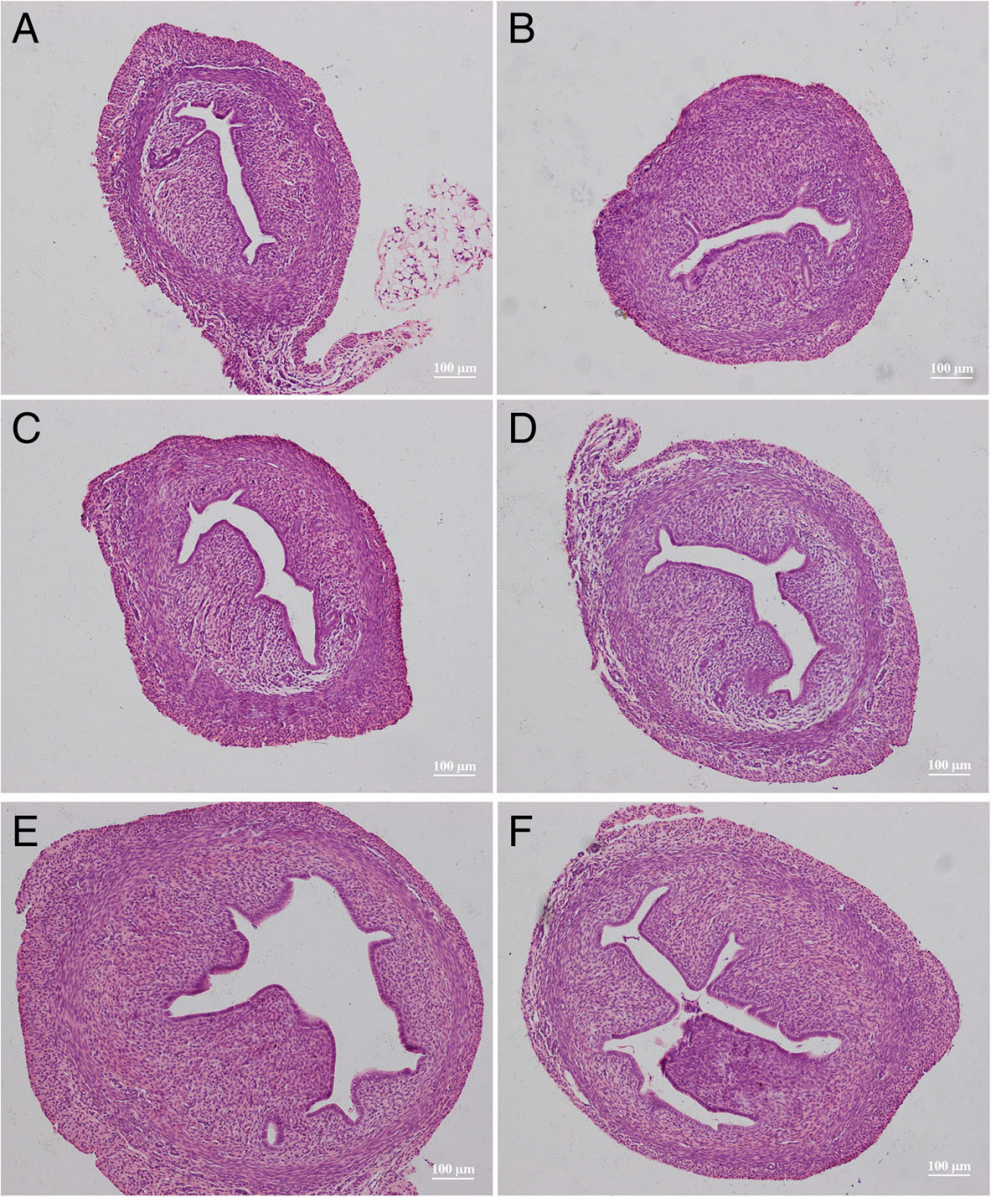
Hematoxylin and eosin stained images of the uterus from solvent control (**a**) and rats receiving BPA dosages of 15 (**b**), 30 (**c**), 60 (**d**), 120 (**e**), and 240 mg/kg body weight (**f**)

**Fig. 5:**
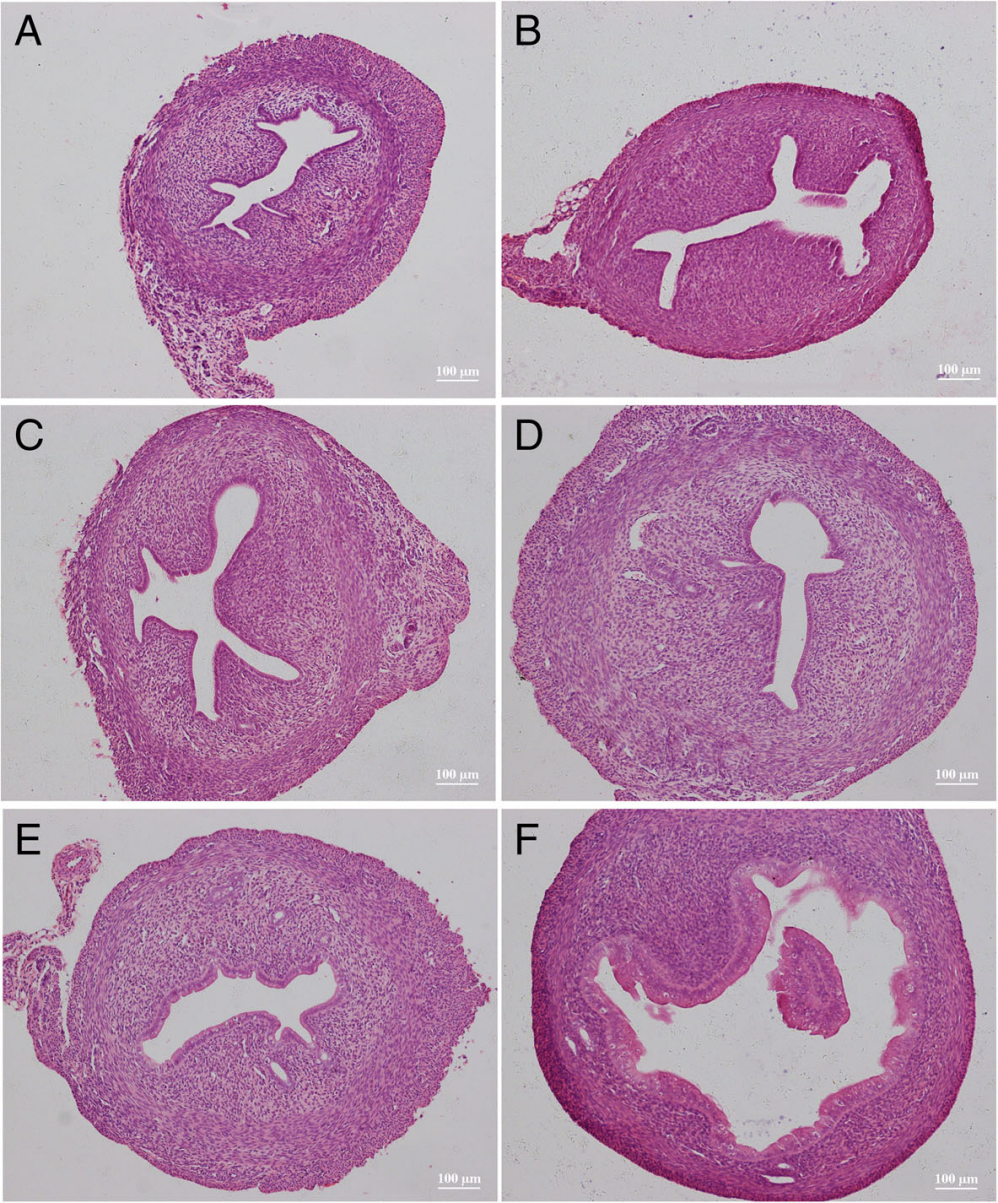
Hematoxylin and eosin stained images of the uterus from solvent control (**a**) and rats receiving NP dosages of 15 (**b**), 30 (**c**), 60 (**d**), 120 (**e**), and 240 mg/kg body weight (**f**)

**Fig. 6:**
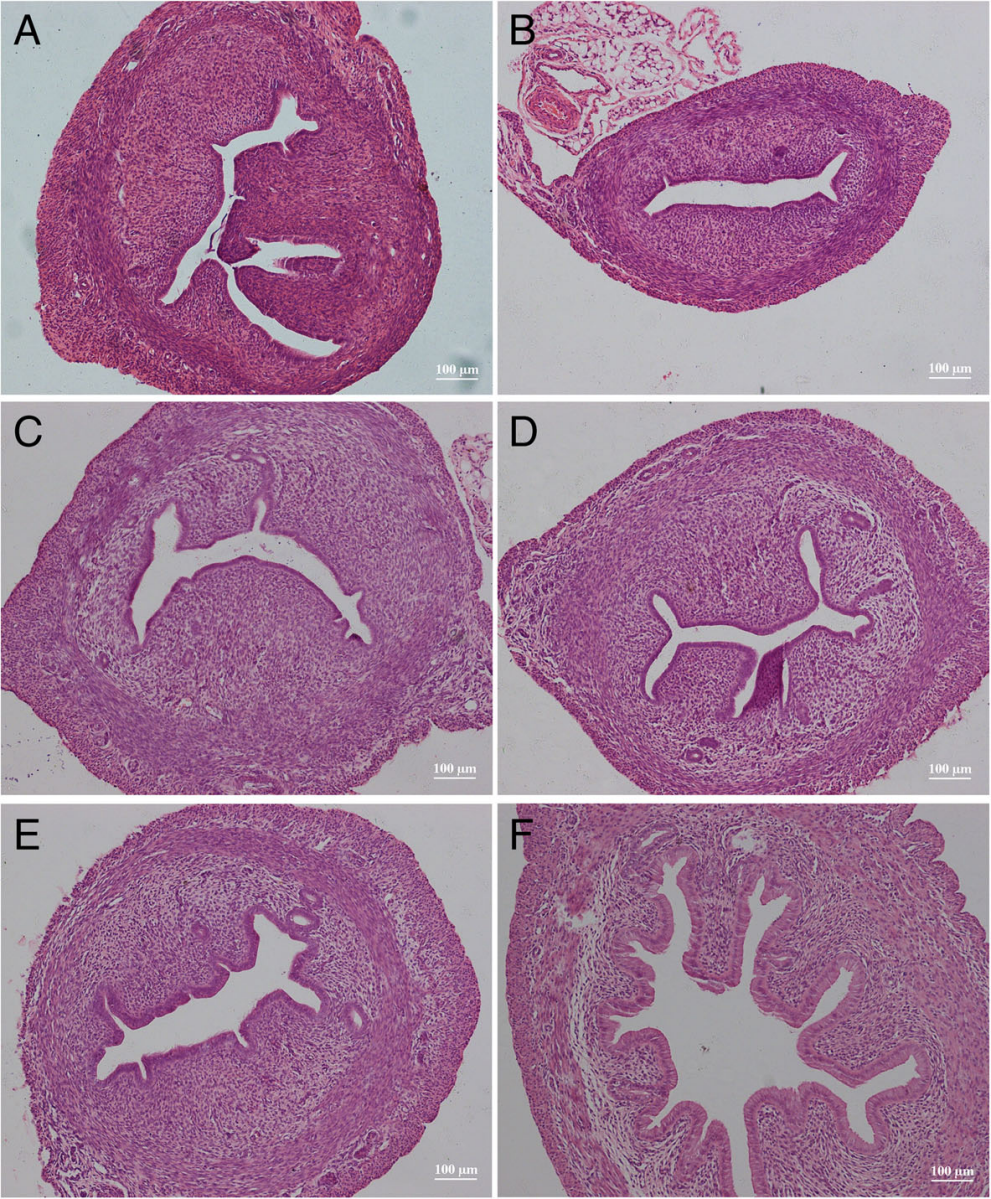
Hematoxylin and eosin stained images of the uterus from solvent control (**a**) and rats receiving DES dosages of 0.25 (**b**), 0.5 (**c**), 1.0 (D), 2.0 (**e**), and 4.0 μg/kg body weight (**f**)

Compared with the solvent control group (Fig. 4a), the uterine cavity was thickened and the cavity area was enlarged in the rats receiving BPA at different dosages (Fig. 4b-f). Especially, increase and enlargement of the uterine glands were observed in groups D and E and endometrial epithelial cells had changed from short columnar cells to tall columnar cells. More significant changes of the uterus were observed in the rats receiving NP (Fig. 5) and DES (Fig. 6). Typical tall columnar epithelial cells covered the endometrium of the rats in the NP groups (Fig. 5d, e) and DES groups (Fig. 6d, e). Most importantly, dramatic changes were evident in the highest dosage groups, including pseudostratified ciliated columnar epithelium, enlarger uterus cavity, and increased numbers of glands (Fig. 5f, 6f). Interestingly, compared with the other groups, DES induced interstitial edema and villi-like changes at the highest dosage. These results showed that NP and DES induced uterus displays the secretory phase earlier. The above results revealed significantly increased the uterus/body weight ratio in treated groups, indicating the ratio is the most sensitive parameter to assess the estrogenic effect.

#### BMD analysis for the uterus/body weight ratio

**Fig. 7:**
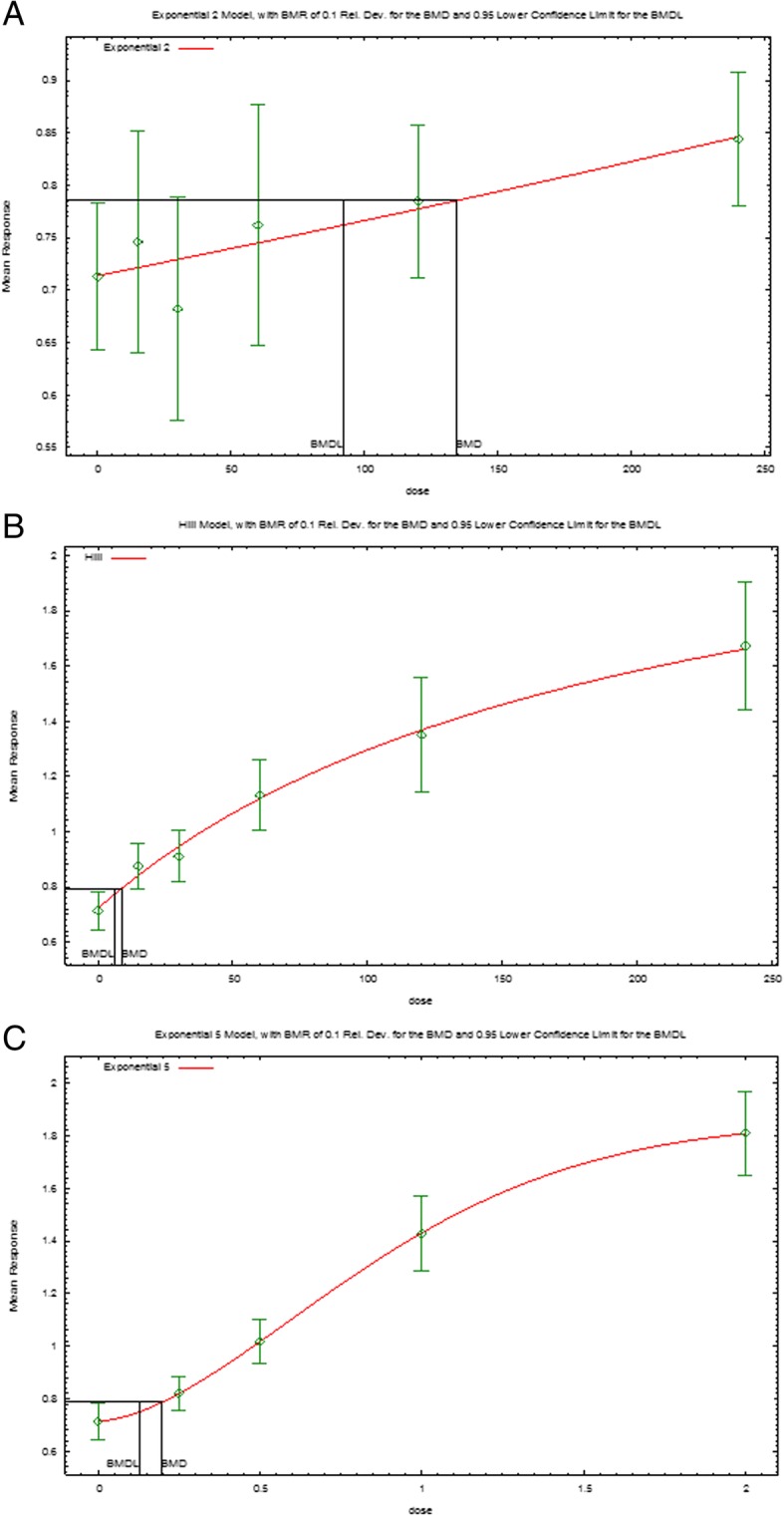
Models of the dose-response relationship of the uterus/body weight ratio simulated for the individual chemicals. **a**. Exponential model for BPA, **b**. Hill model for NP, **c**. Exponential model for DES

Further analyses were necessary for the dose-response modeling of the uterus/body weight ratio of the three EDCs. The analyses used BMD software. Among all models, the Exponential and Hill models were suitable to fit the dose-response curve for the uterus/body weight ratio of the three EDCs. The Exponential model was most suitable for BPA (*p* = 0.72, AIC = -162.82; Fig. 7a) and DES (*p* = 0.98, AIC = -125.12; Fig. 7c), and the Hill model was most suitable for NP (*p* = 0.54, AIC = -128.65; Fig. 7b). The calculated BMDL_10_ values of the uterus/body weight ratio of BPA, NP, and DES were 90, 6, and 0.10 μg/kg body weight, respectively.

### Mixtures toxcity

#### Factorial analysis results

The uterus/body weight ratio was used to conduct factorial analysis. The results are summarized in supporting information Table 2. According to the test of homogeneity of variance, the result reached the homogeneity of variances (*P* > 0.05). Main effect tests indicated no significant change of uterus/body weight ratio for three EDCs at BMDL_10_ doses(*P* > 0.05). At LOAEL doses, the main effect indicated significant changes of uterus/body weight ratio for BPA and DES (*P* < 0.05), but for NP, there was no significant change(*P* > 0.05). For interactions, at BMDL_10_ and LOAEL doses, no inteaction was found for uterus/body weight ratio for BPA, NP and DES(*P* > 0.05). The findings indicated that the combined effect of estrogenic effect is for three EDCs may be additive. The corresponding skeleton map wasn’t used to identify synergistic effect or antagonistic effect because of no interaction effect exists.

**Table 2:** Results of factorial analysis for the relative weight of uterus treated with combination of BPA,NP and DES(*n* = 10)

group	BMDL_10_			LOAEL		
	uterus/body weight ratio (g/kg, %)	*F* Value	*P*-Value	uterus/body weight ratio (g/kg, %)	*F* Value	*P*-Value
Variance source						
Blank control	0.79 ± 0.08	–	–	0.79 ± 0.08	–	*–*
vehicle control	0.75 ± 0.14	–	–	0.75 ± 0.14	–	*–*
Main effect						
BPA	0.80 ± 0.20	3.65	0.06	0.95 ± 0.23	11.87	*P* < 0.05
NP	0.79 ± 0.22	1.00	0.32	0.93 ± 0.18	1.71	0.20
DES	0.85 ± 0.19	0.72	0.40	0.93 ± 0.16	5.57	*P* < 0.05
Interaction						
BPA + NP	0.95 ± 0.27	1.07	0.31	1.00 ± 0.19	1.09	0.30
BPA + DES	0.87 ± 0.21	0.12	0.73	1.08 ± 0.25	0.003	0.96
NP + DES	0.80 ± 0.17	1.07	0.31	0.93 ± 0.12	2.69	0.11
BPA + NP + DES	0.92 ± 0.23	0.01	0.911	1.06 ± 0.11	0.49	0.49

ANOVA was used to test the statistical significance for the main effect and interaction. “*P* < 0.05” indicated statistically significant main effect for individula EDCs or interactions among EDCs

*BPA* Bisphenol A; *NP* Nonylphenol, *DES* Diethylstilbestrol, B*MDL* Benchmark dose lower confidence limit,*LOAEL* Lowest observed adverse effect level

#### CA model analysis results

As shown in Table 3, at BMDL_10_ doses, the MDR range of three EDCs was 0.63–1.93 and all within a factor of 2(0.5 ≤ MDR ≤ 2), the results demosntrated they were consistent with the assumption of addivity. At LOAEL doses, when combined with NP and DES, the MDR was 0.48 and beyond the cutoff poin (0.5 ≤ MDR ≤ 2), indicated it was consistent with the assumption of antagonism. And the other MDR range of three EDCs was 0.54–0.97 and within a factor of 2(0.5 ≤ MDR ≤ 2), the results indicated they were consistent with the assumption of addivity.

**Table 3:** The MDRs between predeiction and tested results of uterus/body weight ratio treated with combination of BPA, NP and DES

group	uterus/body weight ratio (g/kg, %)	weight gain rate of the uterus(%)	predicted effective concentration (mg/kg bw)	MDR	combined effect
BMDL_10_					
BPA + NP	0.95 ± 0.27	26.59 ± 35.85	189.31	1.93	additive
BPA + DES	0.87 ± 0.21	17.11 ± 28.05	119.44	1.30	additive
NP + DES	0.80 ± 0.17	7.21 ± 22.52	3.77	0.63	additive
BPA + NP + DES	0.92 ± 0.23	22.61 ± 31.33	161.82	1.10	additive
LOAEL					
BPA + NP	1.00 ± 0.19	33.53 ± 25.26	250.22	0.97	additive
BPA + DES	1.08 ± 0.25	44.90 ± 32.99	174.08	0.73	additive
NP + DES	0.93 ± 0.12	24.66 ± 15.87	7.21	0.48	antagonistic
BPA + NP + DES	1.06 ± 0.11	41.33 ± 14.81	139.12	0.54	additive

*BPA* Bisphenol A, *NP* Nonylphenol, *DES* Diethylstilbestrol, *BMDL* Benchmark dose lower confidence limit, *LOAEL* Lowest observed adverse effect level

## Discussion

Since EDCs usually exist as mixtures and humans are rarely exposed to a single compound through the environment, foods, and consumer products [26, 27]. This justifies the need to investigate the mixture effects [8]. Food is usually contaminated by low doses of EDCs, such as BPA, NP and DES, through packaging, biological accumulation or manufacturing process [26–28]. Determining human health risks from exposure to chemical mixtures is a daunting challenge to many experimental toxicologists and epidemiologists using observational methods [5]. Therefore, it is necessary to take their combined estrogenic effects into account. It suggests that a simple mixture should be evaluated by testing each individual compound separately, and thereafter different combinations of the compounds [29]. In this study, the mixture of BPA, NP and DES was designed in the equivalent effect of their individual BMDL_10_ and LOAEL values, which were derived from their dose-response curves alone by oral exposure to prepuberty rats. Finally, we present the combined estrogenic effects of the three EDCs based on low doses (BMDL_10_ and LOAEL) by assessing the implications of low-level exposure to multiple chemicals.

In vivo experiments, the rodent uterotropic response assay, long considered the “gold standard” for determining estrogenicity, was identified as a preferred in vivo screen [16, 30] . Many study show the the rat uterotropic assays is a suitable model for testing EDCs for estrogenic activity [31–33]. Here we evaluate the reliability of the 3-days uterotrophic assay for detecting BPA, NP and DES with strong or weak estrogenic activity in 18–20 day rats.

To have a good analysis of the predictability of the combined estrogenic effects of BPA, NP and DES, it is necessary to examine the individual estrogenic effect of each chemical. Our study showed significantly increased the uterus/body weight ratio in treated groups, that indicates the uterus/body weight ratio is the most sensitive parameter to assess the estrogenic effect. From the dose-response relationship of estrogenic effects of BPA, NP and DES in the model of the prepuberty rats, the BMDL_10_(NOAEL) of the estrogenic effects of BPA, NP and DES were 90(120) mg/kg body weight, 6 mg/kg body weight and 0.10(0.25) μg/kg body weight, and the LOAEL of the estrogenic effects of three EDCs were 240 mg/kg body weight, 15 mg/kg body weight and 0.50 μg/kg body weight, respectively, which minimal the mixture combinations at low doses with minimal biological effects. The data in this study are almost consistent with other studies in the literature [6], this leads us to believe that our data are representative of responses seen in rat uterotrophic bioassays.

In order to determine the combination effects of the three EDCs, there is a need for both a better mathematical basis for combination rules that predict effects of mixtures and a fundamental biological concept that supports quantitative formulas for risk assessment of chemical mixtures [34]. Analysis of mixture data is often concerned with comparison to a reference model. There are many models and approaches being introduced to analyze the mixture interactions. Currently, two mathematical models were being developed as a basis for combination rules that predict the adverse effects of mixtures starting from the concentrations of the individual chemicals in the mixture. The most used model is that of concentration addition (CA) which assumes that the combined effect of the two chemicals is the same as it would be if they were both dilutions, of different strength, of the same substance [35]. Another reference model is that of independent action (IA), assuming that the the mixture components act independently of each other and act on different sub-systems (tissues, cells, molecular receptors). Our studies have analyzed the combined estrogenic effects composed of three EDCs that interact with the same sub-system of an organism (uterus). Besides, in experiments with similarly acting chemicals the observed effects were in good agreement with CA predictions [32]. So in such cases, the concept of dose or concentration addition is applicable. In our study, another model of the factorial analysis also was used, which was a classic statistic method that can distinguish differences among a predicting factor, but also can prove if there was interaction between different factors [34]. which model had been used to examine the mixture toxicity of different combined exposure of propylthiouracil, polychlorinated biphenyls, and ammonium perchlorate on thyroid function in the previous studies in our lab [20].

Data from the current study demonstrate that a basically consistent result on mixture toxicity mode at BMDL_10_ doses based on the CA concept and the factorial analysis, the mode of combined effects of the three EDCs were dose addition. Mixtures in LOAEL doses, NP and DES combined effects on rat uterine/body weight ratio indicates antagonistic based on the CA concept but additive based on the factorial analysis. Combined effects of other mixtures are all additive by using the two models. In particular, combined BPA and NP at BMDL_10_ doses that give no statistically significant uterotrophic responses when teste on their own, but when administered together, quite strong ureotrophic effects (*P* < 0.05) are abserved in our test. Good evidence is available to show that combined effects occure even when all mixtures components are present at levels below doses that cause observable effects, the traditional NOAEL/BMDL point of departure for single compound arguably cannot be used to derive a healthbased guidance value for cumulative risk assesement of mixture chemicals. This highlights the deficiencies of the predominant chemical by chemical approach in risk assessment. Mixtures as long as NP and DES coexist, the observed toxicity are less than the the models tested, imply that they may have the antagonistic effect. Causes may be due to the two EDCs have a competition in the same estrogen receptor sites and reduce the estrogenic effects, but the mechanisms of how these chemicals interact with each other on the change of estrogenic effect require further investigations.

For many chemicals, CA will often predict the most conservative mixture effect. CA generally generate slightly more conservative predictions (predicting larger effects than IA), and as databases on chemicals often only provides EC_x_ data or NOAEL or LOAEL which only makes CA predictions possible and not IA, CA is most often the recommended model for risk assessment purposes, irrespective of modes of action [36, 38, 39]. The main drawback of the approach is the use of one global parameter to estimate the compliance or non-compliance between an observed and the CA-predicted mixture toxicity. In the present study, the quantitative comparison of the observed toxicity and the models tested was conducted using the MDR, the deviation from the predictive model were within a factor of two for the CA model, which derived from the frequency distributions of the MDR values of many studies [40]. In our study, the MDR for CA are almost with a factor of 2 ((0.5 ≤ MDR ≤ 2)), only at LOAEL doses, the MDR of NP and DES combined is beyond this cutoff point, and the value is 0.48, very closing to 0.5. From the results, the mixture effects at low doses (BMDL_10_) showed additive effects, while at higher doses (LOAEL) with lower MDR values, the combined effects are to some extend less than additive, which are likely a result of either potential receptor-saturation effects or strong competition among the test compounds for the binding site of the receptors [41]. This study are also in line with those reported on effects of mixtures, non-interaction (additivity) has generally been observed at lower doses of chemicals, whereas the interactions observed were generally at higher doses [41–43]. But this shoud take into account for uncertainty in estimates from the MDR model, the potential deviations from mixture models for error is limited to a particular probability. if the goal is to limit the risk of underestimating toxicity with the CA model to 10%, the MDR is 1.69, and at NOAEL doses, the MDR of BPA and NP combined will exceed this cutoff point, indicating the potential of synergistic interactions [40]. Account for the deviations, it is important to further examine with regard to their toxicity and their frequency of occurrence in the environment.

Compared to the CA model, the factorial analysis was a easily-applied statistical tool, and independence from the toxicological points, the results are easy to read and interpret, but roughly estimate the interaction between variables and when there were multiple factors present, it would be hard to decide where the main effect and interaction came from [37], and it is also hard to quantify the extent of the deviation from the predictive model. The sample size, dose, and sensitivity of endpoints may all affect the iterpretation of interaction mode [20]. In generally, the results of CA and factorial analysis were consistent, but the CA model provides more accurate results than the factorial analysis.

In our study, we performed a more detailed hazard assessment of three EDCs, and selected the POD of each EDCs as the dose design basis for the study of the combined action pattern. It has been shown above that for mixtures, CA can be expected to give a reasonable estimate of the mixture effects, which is intended to give a precautionary estimate of the mixture effects for futher use within risk assessment procedures. But it is still in developemntal stages, the interaction of the mixture largely depended on the concepts and methods used, moreover, problems remained that mixture we tested were with similar modes of action, but low-level presence of various kinds of chemicals with different mechanisms were more likely the case in the environment. So additional studies are needed to further develop predictive models.

## Conclusions

In summary, our objective is to carry out an analysis of interactions of BPA, NP, and DES on estrogenic effects a predefined effect level (BMDL and LOAEL) by using “concentration addition” and “factorial analysis” methods. Our results show that the CA model provides more accurate results than the factorial analysis, the mode of combined effects of the three EDCs were dose addition, except mixtures in LOAEL doses, NP and DES combined effects indicates antagonistic based on the CA concept but additive based on the factorial analysis. In particular, BPA and NP work together to produce combination effects that are larger than the effects of each mixture component applied separately at BMDL doses, clearly show that additivity is important in the assessment of chemicals with estrogenic effects, the use of BMDL as points of departure in risk assessment may lead to underestimations of risk, and a more balanced approach should be considered in risk assessment.

## Abbreviations

ADI: Acceptable daily intake
BMD: Benchmark dose
BMDL: Benchmark dose lower confidence limit
BPA: Bisphenol A
CA: Concentration addition
DES: Diethylstilbestrol
E2: Estradiol
EDC: Endocrine disrupting chemicals
IA: Independent action
LH: Luteinizing hormone
LOAEL: Lowest observed adverse effect level
MDR: Model deviation ratio
NOAEL: No observed adverse effect level
NP: Nonylphenol
OECD: Organization for Economic Co-operation and Development
POD: Point of departure
TDI: Tolerable daily intakes

## References

[1] Colbom T, Dumanoski D, Myers JP (1996). Our stolen future:are we threatening our Fertility,Intelligence,and survival? A scientific detective story.

[2] E D-K, Bourguignon JP, Giudice LC, Hauser R, Prins GS, Soto AM, Zoeller RT, Gore AC (2009). Endocrine-disrupting chemicals: an endocrine society scientific statemen. Endocr Rev.

[3] US EPA. Special report on environmental Endocrine disruption: and effects assessment and analysis ,1997. 10.1289/ehp.98106s111 PMC1533291 9539004

[4] Minatoya M, Itoh S, Yamazaki K, Araki A, Miyashita C, Tamura N, Yamamoto J, Onoda Y, Ogasawara K, Matsumura T, Kishi R (2018). Prenatal exposure to bisphenol a and phthalates and behavioral problems in children at preschool age: the Hokkaido study on environment and Children's health. Environ Health Prev Med.

[5] Gallagher SS, Rice GE, Scarano LJ, Teuschler LK, Bollweg G, Martin L (2015). Cumulative risk assessment lessons learned: a review of case studies and issue papers. Chemosphere..

[6] EFSAScientific opinion on the risks to public health related to the presence of bisphenol a (BPA) in foodstuffs: PART II - toxicological assessment and risk characterization. EFSA J. 2015;13:3978.

[7] An BS, Kang SK, Shin JH, Jeung EB (2002). Stimulation of calbindin-D (9k) mRNA expression in the rat uterus by octyl-phenol, nonylphenol and bisphenol. Molecular & Cellular Endocrinology Molecular & Cellular Endocrinology.

[8] Kortenkamp A (2008). Low dose mixture effects of endocrine disrupters: implications for risk assessment and epidemiology. Int J Androl.

[9] Muncke J (2011). Endocrine disrupting chemicals and other substances of concernin food contact materials: an updated review of exposure, effect and risk assessment. J Steroid Biochem Mol Bio.

[10] Axelstad M, Christiansen S, Boberg J, Scholze M, Jacobsen PR, Isling LK, Kortenkamp A, Hass U (2014). Mixtures of endocrine-disrupting contaminants induce adverse developmental effects in preweaning rats. Reproduction..

[11] Kortenkamp A (2006). Breast cancer, oestrogens and environmental pollutants: are-evaluationfrom a mixture perspective. Int J Androl.

[12] Kortenkamp A (2014). Low dose mixture effects of endocrine disrupters and their implications for regulatory thresholds in chemical risk assessment. Curr Opin Pharmacol.

[13] Newbold RR, Padilla-Banks E, Jefferson WN (2006). Adverse effects of the model environmental estrogen diethylstilbestrol are transmitted to subsequent generations. Endocrinology..

[14] Pacia A, Dołhańczuk-Śródka A (2016). Ziembik Z. Assessment of environmental pollution caused by EDCs from everyday objects.

[15] Kishi R (2017). The Hokkaido birth cohort study on environment and Children's health: cohort profile-updated 2017. Environ Health Prev Med.

[16] Kortenkamp A, Altenburger R (1998). Synergisms with mixtures of xenoestrogens: a reevaluation using the method of isoboles. Sci Total Environ.

[17] EFSA. Human risk assessment of combined exposure to multiple chemicals (2013). EFSA J.

[18] FAO and WHO (2009). Principles and methods for the risk assessment of chemicals in food. Environmental health criteria 240.

[19] OECD (2010). No.440:Uterotrophic bioassay in rodents:a short-term screening test for oestrogenic properties. OECD Guidelines for the Testing of Chemicals.

[20] Chen H, Liu Zhang X, Jia X, Li Q, Su Q, Wang W (2016). Assessment of synergistic thyroid disrupting effects of a mixture of EDCs in ovariectomized rats using factorial analysis and dose addition. Toxicol Res (Camb).

[21] Wang N, Wang XC, Ma X (2015). Characteristics of concentration–inhibition curves of individual chemicals and applicability of the concentration addition model for mixture toxicity prediction. Ecotoxicol Environ Saf.

[22] Junghans M, Backhaus T, Faust M, Scholze M, Grimme LH (2006). Application and validation of approaches for the predictive hazard assessment of realistic pesticide mixtures. Aquat Toxicol.

[23] Christiansen S, Scholze M, Dalgaard M, Vinggaard AM, Axelstad M, Kortenkamp A, Hass U (2009). Synergistic disruption of external male sex organ development by a mixture of four antiandrogens. Environ Health Perspect.

[24] Tollefsen KE, Petersen K, Rowland SJ (2012). Toxicity of synthetic naphthenic acids and mixtures of these to fish liver cells. Environ Sci Technol.

[25] Cedergreen N (2014). Quantifying synergy: a systematic review of mixture toxicity studies within environmental toxicology. PlosOne..

[26] Gan W, Zhou M, Xiang Z, Han X, Li D (2015). Combined effects of nonylphenol and bisphenol a on the human prostate epithelial cell line RWPE-1. Int J Environ Res Pubilc Health.

[27] Jiang X, Chen HQ, Cui ZH, Yin L, Zhang WL, Liu WB, Han F, Ao L, Cao J, Liu JY (2016). Low-dose and combined effects of oral exposure to bisphenol A and diethylstilbestrol on the male reproductive system in adult Sprague-Dawley rats. Environ Toxicol Pharmacol.

[28] von Goetz N, Wormuth M, Scheringer M, Hungerbühler K (2010). Bisphenol a: how the most relevant exposure sources contribute to total consumer exposure. Risk Anal.

[29] Groten JP, Feron VJ, Sühnel J (2001). Toxicology of simple and complex mixtures. Trends Pharmacol Sci.

[30] US EPA. Endocrine Disruptor Screening Program Test Guidelines. OPPTS 890.1600: Uterotrophic Assay. 2009:1–24.

[31] Gray LE, Wilson V, Noriega N, Lambright C, Furr J, Stoker TE, Laws SC, Goldman J, Cooper RL, Foster PM (2004). Use of the laboratory rat as a model in endocrine disruptor screening and testing. ILAR J.

[32] Padilla-Banks E, Jefferson WN, Newbold RR (2001). The immature mouse is a suitable model for detection of estrogenicity in the uterotropic bioassay. Environ Health Perspect.

[33] Reel JR, Lamb JC, Neal BH (1996). Survey and assessment of mammalian estrogen biological assays for hazard characterization. Fundamental & Applied Toxicology.

[34] Hertzberg RC, Teuschler LK (2002). Evaluating quantitative formulas for dose–response assessment of chemical mixtures. Environ Health Perspect.

[35] Yuan Shengwu, Huang Chao, Ji Xiaoya, Ma Mei, Rao Kaifeng, Wang Zijian (2018). Prediction of the combined effects of multiple estrogenic chemicals on MCF-7 human breast cancer cells and a preliminary molecular exploration of the estrogenic proliferative effects and related gene expression. Ecotoxicology and Environmental Safety.

[36] Syberg Kristian, Elleby Anders, Pedersen Henrik, Cedergreen Nina, Forbes Valery E. (2008). Mixture toxicity of three toxicants with similar and dissimilar modes of action to Daphnia magna. Ecotoxicology and Environmental Safety.

[37] Collins LM, Dziak JJ, Kugler KC, Trail JB (2014). Factorial experiments: efficient tools for evaluation of intervention components. Am J Prev Med.

[38] EFSA (2013). Scientific opinion on the relevance of dissimilar mode of action and its appropriate application for cumulative risk assessment of pesticides residues in food. EFSA J.

[39] Backhaus T BH, Faust. Hazard and risk assessment of chemical mixtures under REACH-state of the art, gaps and options for improvement :Swedish chemicals agency,2010. 1–74.

[40] Belden JB, Gilliom RJ, Lydy MJ (2007). How well can we predict the toxicity of pesticide mixtures to aquatic life. Integr Environ Assess Manag.

[41] Ramirez T, Buechse A, Dammann M, Melching-Kollmuß S, Woitkowiak C, van Ravenzwaay B (2014). Effect of estrogenic binary mixtures in the yeast estrogen screen (YES). Regul Toxicol Pharmacol.

[42] Kortenkamp A (2007). Ten Years of Mixing Cocktails: A review of combination effects of endocrine-disrupting chemicals. Environ Health Perspect.

[43] Borgert CJ, Price B, Wells CS, Simon GS (2001). Evaluating chemical interaction studies for mixture risk assessment. Hum Ecol Risk Assess.

